# Gender-related differences in the association between serum uric acid and left ventricular mass index in patients with obstructive hypertrophic cardiomyopathy

**DOI:** 10.1186/s13293-016-0074-x

**Published:** 2016-04-05

**Authors:** Changlin Zhang, Rong Liu, Jiansong Yuan, Jingang Cui, Fenghuan Hu, Weixian Yang, Yan Zhang, Chengzhi Yang, Shubin Qiao

**Affiliations:** Department of Cardiology, State Key Laboratory of Cardiovascular Disease, Fuwai Hospital, National Center for Cardiovascular Disease, Chinese Academy of Medical Sciences and Peking Union Medical College, No.167 Beilishi Road, Xicheng District, Beijing, 100037 China; Department of Radiology, State Key Laboratory of Cardiovascular Disease, Fuwai Hospital, National Center for Cardiovascular Disease, Chinese Academy of Medical Sciences and Peking Union Medical College, No.167 Beilishi Road, Xicheng District, Beijing, 100037 China

**Keywords:** Gender difference, Uric acid, Left ventricular mass index, Obstructive hypertrophic cardiomyopathy

## Abstract

**Background:**

Serum uric acid (SUA) is associated with left ventricular hypertrophy in a wide spectrum of study population. However, whether this association exists in patients with hypertrophic cardiomyopathy (HCM, including obstructive HCM), and if present, whether gender has any impact on this association, remains unknown.

**Methods:**

A total of 161 patients with obstructive HCM (age 47.2 ± 10.8 years, 99 (62 %) men) were included in this study. All patients underwent extensive clinical, laboratory, echocardiographic, and cardiac magnetic resonance (CMR) imaging examinations. Left ventricular mass index (LVMI) was assessed using CMR.

**Results:**

The mean value of SUA was 353.4 ± 87.5 μmol/L. Both SUA levels (381.2 ± 86.4 vs. 309.0 ± 69.3 μmol/L, *p* < 0.001) and LVMI (96.2 ± 32.1 vs. 84.4 ± 32.4 g/m^2^, *p* = 0.025) were significantly higher in men than in women. LVMI increased progressively across sex-specific tertiles of SUA in women (*p* = 0.030), but not in men (*p* = 0.177). SUA was positively correlated with LVMI in female patients (*r* = 0.372, *p* = 0.003), but not in males (*r* = 0.112, *p* = 0.269). On multivariate linear regression analysis, SUA was independently associated with LVMI in females (*β* = 0.375, *p* = 0.002), but not in males.

**Conclusions:**

SUA levels are significantly and independently associated with LVMI in women with obstructive HCM, but not in men. Our findings imply the potential significance of urate-lowering regimens in female patients with obstructive HCM.

## Background

Serum uric acid (SUA), as an end metabolite of purine degradation, is associated with multiple established cardiovascular risk factors, including hypertension, diabetes mellitus, dyslipidemia, obesity, and metabolic syndrome [1–5]. Although its importance in cardiovascular conditions remains controversial [6], elevated SUA has been shown to be an independent predictor for the incidence and adverse cardiovascular outcomes of coronary heart disease, myocardial infarction, heart failure, stroke, atrial fibrillation, and chronic kidney disease (CKD) [7–12]. Baseline SUA levels are also related to an increased risk for cardiovascular and all-cause mortality in the general population [13]. Multiple studies, although not all, have demonstrated that SUA levels are significantly and independently associated with left ventricular hypertrophy (LVH) and left ventricular mass index (LVMI) in the general population and in patients with essential hypertension, CKD, and renal transplant [14–24]. Moreover, divergent sex differences in the relationship between SUA and LVH have been observed. Some studies found this relationship only present in males [14, 16, 18, 25], while others only in females [17, 20, 22].

Hypertrophic cardiomyopathy (HCM) is a common inherited heart disease, mainly with an autosomal dominant pattern, and caused by more than 1500 individual mutations in 11 or more genes encoding cardiac sarcomere proteins [26, 27]. It can lead to heterogeneous clinical presentations, ranging from asymptomatic status with normal longevity to progressive heart failure, atrial fibrillation with risk for embolic stroke, and sudden death [27, 28]. HCM is characterized by unexplained LVH, as well as myocyte disarray, replacement fibrosis, and small vessel disease [29]. Quite recently, uric acid has been reported to be independently associated with adverse prognosis in patients with HCM [30]. However, the pathophysiological mechanisms underlying this association have not been elucidated. There are scanty data regarding the relationship between SUA and LVH in patients with HCM [30]. In addition, it is well known that the majority of HCM patients (about 70 %) have left ventricular outflow tract (LVOT) obstruction present at rest or with physiologic exercise [31]. Accordingly, the aim of our study was to examine in patients with obstructive HCM the association between SUA and LVMI (as assessed by cardiac magnetic resonance (CMR) imaging) and to evaluate whether gender has any impact on this association.

## Methods

### Patients

Between November 2008 and August 2013, consecutive patients with obstructive HCM who were referred to Fuwai Hospital (Beijing, China) for a comprehensive cardiac evaluation were enrolled in this study. HCM was diagnosed by an otherwise unexplained increase in left ventricular wall thickness (≥15 or ≥13 mm with a definite family history of HCM), as determined with echocardiography or CMR [26]. LVOT obstruction was considered as a peak instantaneous LVOT pressure gradient ≥30 mmHg at rest or during physiological provocation such as Valsalva maneuver, standing, and exercise [26]. A complete evaluation was performed in each enrolled patient, including demographic characteristics, medical history, physical examination, 12-lead electrocardiography (ECG), 24-h ambulatory ECG monitoring, transthoracic echocardiography (TTE), routine blood tests, CMR, and coronary angiography. Patients with the following conditions were excluded from the present study: significant coronary artery disease (epicardial coronary stenosis >70 % on coronary angiography, previous myocardial infarction, bypass surgery, or percutaneous coronary intervention), valvular heart disease, left ventricular ejection fraction (LVEF) <50 % as measured by echocardiography or CMR, cerebrovascular disease, renal dysfunction (defined as an estimated glomerular filtration rate (eGFR) <60 mL/min/1.73 m^2^), gout, concomitant neoplasm, infection, connective tissue disease, pregnancy, or breast feeding. None of the included patients were receiving any urate-lowering agents. Patients with a history of alcohol septal ablation, septal myectomy, or permanent mechanical device implantation were also excluded. Eventually, 161 patients were included in the current study.

The study protocol was approved by the Ethics Committee of Fuwai Hospital and conducted in accordance with the Declaration of Helsinki. All patients gave their written informed consent.

### Echocardiography

TTE was performed by an experienced cardio-sonographer using the Phillips iE33 Color Doppler Ultrasound System (Philips Healthcare, Andover, MA, USA). M-mode, two-dimensional, and pulsed and continuous-wave Doppler studies were utilized in the standard evaluation according to the guidelines of the American Society of Echocardiography [32]. All patients underwent resting LVOT gradient measurements with continuous-wave Doppler echocardiography, while LVOT gradient after provocation was only determined in those with a resting LVOT gradient <50 mmHg. The severity of mitral regurgitation (MR), graded from mild to severe in accordance with European association of echocardiography criteria, was evaluated semi-quantitatively using color Doppler flow imaging [33].

### CMR protocol and image analysis

CMR imaging was performed on a 1.5-T scanner (Magnetom Avanto, Siemens Medical Solutions, Erlangen, Germany) with electrocardiographic gating and breath holding, as described in detail previously [34, 35]. Briefly, cine images were obtained by a true fast imaging with steady-state precession (TrueFisp) sequence, in left ventricular (LV) two-chamber and four-chamber long-axis views, LVOT view, and LV short-axis views (contiguous slices from the apex to base for the full coverage of LV). The following imaging parameters were used: slice thickness 6 mm, repetition time 2.7 ms, echo time 1.2 ms, field of view 360 × 315 mm^2^, flip angle 70°, temporal resolution 40 ms, and image matrix 192 × 162.

All CMR images were analyzed off-line by an experienced radiologist, using dedicated software (version VE36A, ARGUS, Siemens, Germany) for image analysis. The LV endocardial and epicardial contours were manually planimetried at end diastole and end systole on each short-axis slice, taking special care to exclude the papillary muscles from the total myocardial mass. LV volumes, LVEF, and LV mass (LVM) were then derived in a standard manner. LVM was calculated from the LV myocardial volume (measured at end diastole) multiplied by the specific gravity of myocardium (1.05 g/mL). All of those measurements were indexed to body surface area (BSA) in square meter, except LVEF. The LV end-diastolic diameter (LVEDD) and maximal wall thickness were determined on the short-axis cine images at end diastole.

### Biochemical measurements

Venous blood samples were taken in the early morning after an overnight fast, within 2 days of TTE and 1 week of CMR examination. All of those samples were sent to the clinical laboratory of Fuwai hospital immediately and analyzed by medical technologists who were unaware of any clinical information about the study patients. Serum levels of creatinine, uric acid, total cholesterol (TC), triglycerides (TG), high-density lipoprotein cholesterol (HDL-C), low-density lipoprotein cholesterol (LDL-C), fasting blood glucose (FBG), and high-sensitivity C-reactive protein (hs-CRP) were measured by routine laboratory methods using an Olympus AU-5400 auto-analyzer (Olympus Corporation, Mishama, Japan). SUA concentrations were determined using the uricase colorimetric assay (Uric Acid Kit, Biosino, Beijing, China). Plasma levels of N-terminal pro-B-type natriuretic peptide (NT-proBNP) were also measured, with an electrochemiluminescent immunoassay (Elecsys proBNP II assay, Roche Diagnostics GmbH, Mannheim, Germany) on a Cobas 6000 analyzer (Roche Diagnostics). The eGFR (mL/min/1.73 m^2^) was calculated using the Chronic Kidney Disease Epidemiology Collaboration (CKD-EPI) equation [36]. Dyslipidemia was defined as those with serum LDL-C ≥3.37 mmol/L, TG ≥1.70 mmol/L, HDL-C <1.04 mmol/L, or the use of lipid-lowering drugs.

### Statistical analysis

Continuous variables are presented as either mean ± standard deviation (SD) or median (interquartile range (IQR)), according to their distribution. Categorical variables are shown as frequencies (percentages). Differences of continuous variables were assessed with the independent Student’s *t* test, Mann–Whitney *U* test, one-way analysis of variance (followed by the Dunnett’s *t* test for multiple comparisons) or Kruskal–Wallis H test (as appropriate). Categorical variables were compared using the *χ*^2^ test or Fisher’s exact test (as appropriate). The simple correlation between two continuous variables was examined using the Pearson or Spearman correlation test (as appropriate). To evaluate if SUA was an independent risk factor for LVMI in our patients with obstructive HCM, stepwise multiple linear regression analysis (*p* value threshold to enter ≤0.05; to remove, ≤0.10) was applied by adjusting for potential confounding factors affecting LVMI. All data analyses were undertaken using the statistical package SPSS 21.0 (SPSS Inc, Chicago, IL, USA). A two-tailed *p* < 0.05 was considered statistically significant.

## Results

The mean age of the 161 obstructive HCM patients was 47.2 ± 10.8 years (range, 20–71 years), and there were 99 males and 62 females. Most (86 %) of them had moderate or severe dyspnea (NYHA functional class III/IV). Mean resting LVOT gradient was 78.6 ± 32.4 mmHg. The concentrations of SUA ranged from 155.9 to 648.1 μmol/L (mean, 353.4 ± 87.5 μmol/L). The demographic and clinical parameters of those patients, analyzed on the basis of gender, are presented in Table 1. Men were younger (45.4 ± 10.0 vs. 50.1 ± 11.5 years, *p* = 0.008) and more likely to smoke (58 vs. 3 %, *p* < 0.001) than women. As expected, SUA levels (381.2 ± 86.4 vs. 309.0 ± 69.3 μmol/L, *p* < 0.001) and LVMI (96.2 ± 32.1 vs. 84.4 ± 32.4 g/m^2^, *p* = 0.025) were significantly higher in men than in women. In addition, compared with women, men had significantly higher body mass index (BMI), left ventricular end-diastolic diameter (LVEDD) and LVM, and lower levels of HDL-C and NT-proBNP. The values of eGFR (101.7 ± 12.8 vs. 99.8 ± 14.6 mL/min/1.73 m^2^, *p* = 0.386) were comparable between the two genders, although serum creatinine (77.2 ± 11.3 vs. 60.1 ± 11.2 μmol/L, *p* < 0.001) was greater in men than in women.

**Table 1:** Demographic and clinical characteristics of the obstructive hypertrophic cardiomyopathy patients according to gender

Variable	Male (*n* = 99)	Female (*n* = 62)	*p* value
Age (years)	45.4 ± 10.0	50.1 ± 11.5	0.008
Body mass index (kg/m^2^)	26.3 ± 3.4	24.6 ± 3.0	0.004
Hypertension	26 (26 %)	22 (36 %)	0.213
Diabetes mellitus	5 (5 %)	1 (2 %)	0.488
Dyslipidemia	32 (32 %)	15 (24 %)	0.27
Current smokers	57 (58 %)	2 (3 %)	<0.001
Duration of obstructive HC (months)	12 (2–48)	12 (2–48)	0.729
NYHA functional class			0.636
I	4 (4 %)	5 (8 %)	
II	10 (10 %)	4 (7 %)	
III	71 (72 %)	41 (66 %)	
IV	14 (14 %)	12 (19 %)	
Syncope	30 (30 %)	23 (37 %)	0.372
Family history of HC	20 (20 %)	16 (26 %)	0.406
Family history of sudden death	11 (11 %)	6 (10 %)	0.773
Systolic blood pressure (mmHg)	117.2 ± 16.1	117.5 ± 19.3	0.902
Diastolic blood pressure (mmHg)	73.8 ± 11.1	71.6 ± 11.2	0.226
Heart rate (beats/min)	68.6 ± 9.9	70.8 ± 12.8	0.252
Atrial fibrillation	7 (7 %)	6 (10 %)	0.555
Non-sustained ventricular tachycardia^a^	4 (5 %)	5 (10 %)	0.433
Medications			
Beta-blockers	79 (80 %)	45 (73 %)	0.289
Calcium antagonists	26 (26 %)	25 (40 %)	0.062
Amiodarone	5 (5 %)	2 (3 %)	0.877
ACEI/ARB	11 (11 %)	14 (23 %)	0.051
Statins	10 (10 %)	8 (13 %)	0.583
Aspirin	25 (25 %)	15 (24 %)	0.880
Diuretics	4 (4 %)	5 (8 %)	0.466
Laboratory data			
Serum creatinine (μmol/L)	77.2 ± 11.3	60.1 ± 11.2	<0.001
eGFR (mL/min/1.73 m^2^)	101.7 ± 12.8	99.8 ± 14.6	0.386
Blood glucose (mmol/L)	4.85 ± 0.59	4.85 ± 0.62	0.978
HbA1c (%)	5.59 ± 0.53	5.60 ± 0.52	0.993
Total cholesterol (mmol/L)	4.62 ± 1.01	4.54 ± 1.00	0.604
HDL cholesterol (mmol/L)	1.07 ± 0.25	1.26 ± 0.30	<0.001
LDL cholesterol (mmol/L)	2.89 ± 0.92	2.76 ± 0.75	0.380
Triglycerides (mmol/L)	1.69 ± 0.93	1.52 ± 0.83	0.251
hs-CRP (mg/L)	1.25 (0.60–2.14)	0.96 (0.40–1.94)	0.318
NT-proBNP (pmol/L)	1154.1 (775.5–1692.5)	1811.9 (869.6–2816.0)	0.002
Serum uric acid (μmol/L)	381.2 ± 86.4	309.0 ± 69.3	<0.001
Echocardiography			
Systolic anterior motion	94 (95 %)	58 (94 %)	0.981
Moderate or severe mitral regurgitation	36 (36 %)	25 (40 %)	0.614
LVOTG at rest (mmHg)	77.9 ± 32.5	79.7 ± 32.5	0.735
LVOTG after provocation (mmHg)^b^	95.7 ± 28.9	87.8 ± 15.8	0.312
LVOTG at rest ≥30 mmHg	93 (94 %)	57 (92 %)	0.865
Cardiac magnetic resonance			
Left atrium diameter (mm)	39.8 ± 7.6	40.7 ± 7.9	0.483
LV end-diastolic diameter (mm)	46.4 ± 4.1	44.7 ± 4.1	0.010
Maximum wall thickness (mm)	23.5 ± 4.4	23.0 ± 5.4	0.543
Maximum wall thickness ≥30 mm	8 (8 %)	5 (8 %)	0.997
LV ejection fraction (%)	72.2 ± 7.2	72.0 ± 7.3	0.893
LV mass (g)	180.6 ± 64.9	137.4 ± 53.3	<0.001
LV end-diastolic volume index (mL/m^2^)	65.1 ± 14.1	63.9 ± 13.9	0.61
LV end-systolic volume index (mL/m^2^)	18.1 ± 6.1	18.1 ± 7.0	0.976
Stroke volume index (mL/m^2^)	47.0 ± 11.3	45.8 ± 9.6	0.496
Cardiac index (L/min/m^2^)	3.12 ± 0.77	3.13 ± 0.81	0.925
LV mass index (g/m^2^)	96.2 ± 32.1	84.4 ± 32.4	0.025

Data are expressed as mean ± SD, number (percentage), or median (interquartile range)

*ACEI* angiotensin-converting enzyme inhibitor, *ARB* angiotensin receptor blocker, *eGFR* estimated glomerular filtration rate, *HbA1c* glycated hemoglobin, *HC* hypertrophic cardiomyopathy, *HDL* high-density lipoprotein, *hs-CRP* high-sensitivity C-reactive protein, *LDL* low-density lipoprotein, *LV* left ventricular, *LVOTG* left ventricular outflow tract gradient, *NT-proBNP* N-terminal pro-B-type natriuretic peptide, *NYHA* New York Heart Association

^a^Ambulatory 24-h Holter monitoring results were available in 127 of the 161 study patients

^b^LVOTG was provoked in 50 of the 161 study patients

**Fig. 1:**
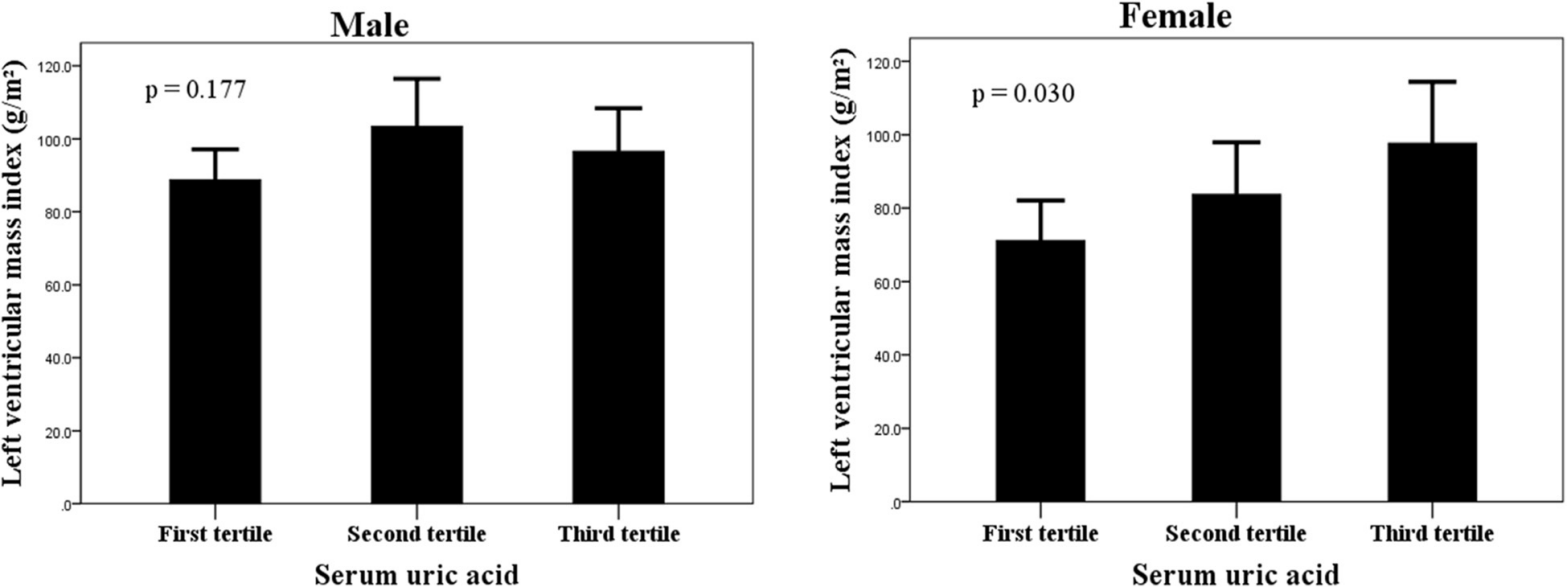
Left ventricular mass index across sex-specific tertiles of serum uric acid in men and women. *p* values are for differences across tertiles of serum uric acid in each gender

The demographic and clinical data of the study patients on the basis of sex-specific tertiles of SUA are reported in Table 2. In male patients, BMI (*p* = 0.022), serum TG (*p* = 0.001), and left atrial (LA) diameter (*p* = 0.025) increased significantly with ascending SUA tertiles. Men in the third tertile had significantly lower resting LVOT gradient than those in the second tertile (*p* < 0.05). Compared with the first and second tertiles, the prevalence of resting LVOT gradient ≥30 mmHg was significantly lower in the third tertile (*p* = 0.003). Of note, LVMI did not differ significantly across SUA tertiles in men (*p* = 0.177), whereas it did show a progressive increase with growing tertiles of SUA in women (*p* = 0.030, Fig. 1). There were no significant differences with respect to age, eGFR, hs-CRP, NT-proBNP, medications taken (including loop and thiazide diuretics), maximal wall thickness (MWT), LVM and the prevalence of hypertension, diabetes mellitus, smoking, and dyslipidemia among the SUA tertiles in both sexes. BMI (*p* = 0.820), resting LVOT gradient (*p* = 0.398), and the percentage of menopause (*p* = 0.863) were similar across tertiles of SUA in females.

**Table 2:** Demographic and clinical characteristics of the obstructive hypertrophic cardiomyopathy patients according to gender-specific tertiles of serum uric acid

	Male				Female			
Variable	First tertile (*n* = 33)	Second tertile (*n* = 33)	Third tertile (*n* = 33)	*p* value	First tertile (*n* = 20)	Second tertile (*n* = 21)	Third tertile (*n* = 21)	*p* value
Serum uric acid range (μmol/L)	<342.7	342.7–410.8	≥410.8		<266.3	266.3–328.2	≥328.2	
Age (years)	45.2 ± 10.1	45.9 ± 10.8	45.2 ± 9.4	0.959	48.5 ± 12.9	50.9 ± 9.4	50.8 ± 12.5	0.755
Body mass index (kg/m^2^)	25.1 ± 3.5	26.0 ± 3.3	27.4 ± 2.9	0.022	25.0 ± 3.0	24.4 ± 2.6	24.5 ± 3.4	0.820
Menopause	–	–	–	–	9 (45 %)	11 (52 %)	11 (52 %)	0.863
Hypertension	8 (24 %)	11 (33 %)	7 (21 %)	0.508	6 (30 %)	8 (38 %)	8 (38 %)	0.824
Diabetes mellitus	3 (9 %)	0 (0 %)	2 (6 %)	0.365	0 (0 %)	1 (5 %)	0 (0 %)	1.000
Dyslipidemia	8 (24 %)	10 (30 %)	14 (42 %)	0.274	3 (15 %)	5 (24 %)	7 (33 %)	0.408
Current smokers	18 (55 %)	18 (55 %)	21 (64 %)	0.689	0 (0 %)	1 (5 %)	1 (5 %)	1.000
Duration of obstructive HC (months)	7 (1.5–42)	12 (2–66)	12 (1.5–60)	0.435	12 (1.25–36)	12 (1.5–24)	12 (3–72)	0.562
NYHA class III/IV	27 (82 %)	30 (91 %)	28 (85 %)	0.672	16 (80 %)	19 (91 %)	18 (86 %)	0.610
Syncope	10 (30 %)	8 (24 %)	12 (36 %)	0.563	6 (30 %)	7 (33 %)	10 (48 %)	0.459
Family history of HC	8 (24 %)	9 (27 %)	3 (9 %)	0.143	8 (40 %)	4 (19 %)	4 (19 %)	0.212
Family history of sudden death	5 (15 %)	4 (12 %)	2 (6 %)	0.614	4 (20 %)	0 (0 %)	2 (10 %)	0.073
Systolic blood pressure (mmHg)	119.8 ± 16.9	118.3 ± 17.7	113.5 ± 13.2	0.256	114.5 ± 13.5	120.5 ± 20.7	117.5 ± 22.9	0.616
Diastolic blood pressure (mmHg)	73.9 ± 10.9	74.8 ± 12.5	72.7 ± 9.9	0.742	69.3 ± 8.6	75.1 ± 12.9	70.4 ± 11.1	0.257
Heart rate (beats/min)	67.8 ± 9.5	69.1 ± 10.6	68.8 ± 9.7	0.861	72.4 ± 14.1	66.6 ± 8.7	73.5 ± 14.4	0.147
Atrial fibrillation	0 (0 %)	3 (9 %)	4 (12 %)	0.156	2 (10 %)	1 (5 %)	3 (14 %)	0.682
Non-sustained ventricular tachycardia^a^	2 (8 %)	1 (4 %)	1 (4 %)	0.836	1 (6 %)	1 (6 %)	3 (21 %)	0.414
Medications								
Beta-blockers	26 (79 %)	30 (91 %)	23 (70 %)	0.098	14 (70 %)	18 (86 %)	13 (62 %)	0.213
Calcium antagonists	12 (36 %)	10 (30 %)	4 (12 %)	0.066	5 (25 %)	9 (43 %)	11 (52 %)	0.194
Amiodarone	2 (6 %)	1 (3 %)	2 (6 %)	1.000	0 (0 %)	1 (5 %)	1 (5 %)	1.000
ACEI/ARB	6 (18 %)	2 (6 %)	3 (9 %)	0.370	4 (20 %)	4 (19 %)	6 (29 %)	0.801
Statins	2 (6 %)	6 (18 %)	2 (6 %)	0.208	1 (5 %)	3 (14 %)	4 (19 %)	0.508
Aspirin	5 (15 %)	9 (27 %)	11 (33 %)	0.223	6 (30 %)	3 (14 %)	6 (29 %)	0.498
Diuretics	0 (0 %)	3 (9 %)	1 (3 %)	0.320	2 (10 %)	1 (5 %)	2 (10 %)	0.864
Laboratory data								
Serum creatinine (μmol/L)	73.7 ± 12.1	79.5 ± 10.0	78.4 ± 11.2	0.089	59.1 ± 12.5	59.9 ± 12.8	61.3 ± 8.5	0.824
eGFR (mL/min/1.73 m^2^)	104.4 ± 11.1	99.7 ± 13.6	101.2 ± 13.3	0.313	101.5 ± 17.0	98.5 ± 13.7	99.6 ± 13.4	0.805
Fasting blood glucose (mmol/L)	4.77 ± 0.70	4.85 ± 0.46	4.94 ± 0.58	0.509	4.96 ± 0.55	4.82 ± 0.82	4.78 ± 0.43	0.645
HbA1c (%)	5.56 ± 0.40	5.55 ± 0.54	5.68 ± 0.63	0.528	5.45 ± 0.26	5.69 ± 0.78	5.64 ± 0.35	0.316
Total cholesterol (mmol/L)	4.34 ± 0.73	4.68 ± 1.18	4.85 ± 1.03	0.108	4.21 ± 0.89	4.76 ± 0.96	4.62 ± 1.09	0.186
HDL cholesterol (mmol/L)	1.13 ± 0.24	1.07 ± 0.28	1.01 ± 0.20	0.144	1.26 ± 0.37	1.23 ± 0.21	1.29 ± 0.32	0.814
LDL cholesterol (mmol/L)	2.61 ± 0.71	3.00 ± 1.04	3.05 ± 0.95	0.102	2.43 ± 0.71	2.94 ± 0.77	2.90 ± 0.70	0.056
Triglycerides (mmol/L)	1.36 ± 0.58	1.52 ± 0.64	2.18 ± 1.23	0.001	1.34 ± 0.82	1.62 ± 0.78	1.61 ± 0.91	0.476
hs-CRP (mg/L)	1.11 (0.30–2.12)	1.26 (0.69–1.83)	1.36 (0.88–2.99)	0.291	1.04 (0.39–1.78)	0.81 (0.28–1.65)	1.67 (0.68–2.96)	0.111
NT-proBNP (pmol/L)	1140.9 (863.9–1459.1)	1314.9 (812.5–2089.4)	1154.1 (699.6–1578.7)	0.536	1518.2 (862.2–2670.2)	1392.5 (833.6–2558.0)	2308.2 (1205.9–2966.6)	0.299
Serum uric acid (μmol/L)	292.5 ± 42.8	373.3 ± 19.1	477.6 ± 54.0	<0.001	236.4 ± 22.0	298.2 ± 13.0	388.9 ± 44.0	<0.001
Echocardiography								
Systolic anterior motion	30 (91 %)	33 (100 %)	31 (100 %)	0.365	18 (90 %)	20 (95 %)	20 (95 %)	0.684
Moderate or severe MR	13 (39 %)	12 (36 %)	11 (33 %)	0.877	10 (50 %)	7 (33 %)	8 (38 %)	0.536
LVOTG at rest (mmHg)	78.1 ± 27.5	89.7 ± 34.4	66.0 ± 31.6	0.011	81.3 ± 33.5	85.7 ± 32.1	72.2 ± 32.1	0.398
LVOTG at rest ≥30 mmHg	33 (100 %)	33 (100 %)	27 (82 %)	0.003	18 (90 %)	20 (95 %)	19 (91 %)	0.864
LVOTG after provocation (mmHg)^b^	96.2 ± 35.9	98.9 ± 21.6	91.7 ± 30.2	0.843	90.6 ± 8.0	88.3 ± 20.7	85.6 ± 18.8	0.877
Cardiac magnetic resonance								
Left atrium diameter (mm)	37.8 ± 6.6	39.1 ± 8.3	42.6 ± 7.2	0.025	39.6 ± 8.6	38.8 ± 6.5	43.8 ± 8.0	0.088
LV end-diastolic diameter (mm)	46.8 ± 3.6	46.2 ± 5.2	46.3 ± 3.4	0.828	45.1 ± 4.5	45.6 ± 3.8	43.4 ± 3.9	0.213
Maximum wall thickness (mm)	23.1 ± 3.6	24.3 ± 5.6	23.0 ± 3.6	0.449	23.1 ± 3.5	21.9 ± 4.3	24.0 ± 7.4	0.429
Maximum wall thickness ≥30 mm	1 (3 %)	5 (15 %)	2 (6 %)	0.266	1 (5 %)	1 (5 %)	3 (14 %)	0.605
LV ejection fraction (%)	71.5 ± 7.5	72.7 ± 4.6	72.4 ± 8.9	0.775	72.1 ± 7.8	71.2 ± 7.1	72.7 ± 7.3	0.805
LV mass (g)	162.5 ± 45.2	194.4 ± 77.8	185.0 ± 65.2	0.122	117.6 ± 37.5	136.6 ± 50.5	157.0 ± 63.0	0.058
LV end-diastolic volume index (mL/m^2^)	65.2 ± 12.3	66.2 ± 15.1	63.9 ± 15.1	0.797	60.1 ± 10.0	69.8 ± 16.4	61.8 ± 13.0	0.053
LV end-systolic volume index (mL/m^2^)	18.7 ± 6.7	17.9 ± 4.6	17.7 ± 7.0	0.786	16.9 ± 7.3	20.4 ± 7.6	16.9 ± 5.8	0.177
Stroke volume index (mL/m^2^)	46.5 ± 9.4	48.3 ± 12.0	46.2 ± 12.6	0.723	43.2 ± 6.9	49.3 ± 10.7	44.8 ± 9.9	0.100
Cardiac index (L/min/m^2^)	3.16 ± 0.69	3.16 ± 0.92	3.02 ± 0.70	0.709	2.99 ± 0.61	3.31 ± 0.87	3.07 ± 0.89	0.398
LV mass index (g/m^2^)	88.7 ± 23.7	103.4 ± 37.0	96.6 ± 33.4	0.177	71.2 ± 23.1	83.7 ± 31.3	97.7 ± 36.9	0.030

Data are expressed as mean ± SD, number (percentage), or median (interquartile range)

*MR* mitral regurgitation and other abbreviations as in Table 1

^a^Ambulatory 24-h Holter monitoring results were available in 127 of the 161 study patients

^b^LVOTG was provoked in 50 of the 161 study patients

**Fig. 2:**
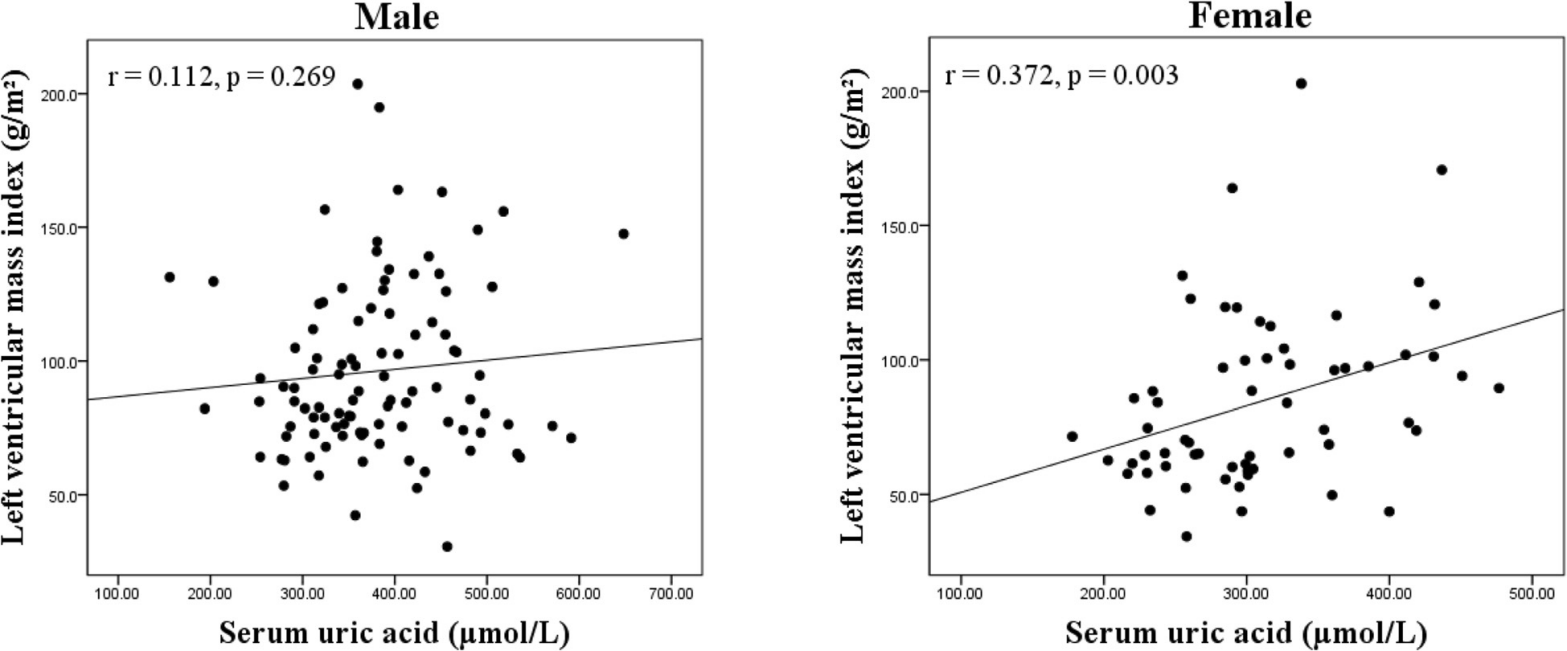
Scatter plots showing the correlations between serum uric acid and left ventricular mass index in each gender

In males, SUA was positively correlated with BMI (*r* = 0.309, *p* = 0.002), TG (*r* = 0.343, *p* = 0.001), hs-CRP (*r* = 0.198, *p* = 0.049), and LA diameter (*r* = 0.247, *p* = 0.014), while negatively with HDL-C (*r* = −0.259, *p* = 0.010; Table 3). No significant correlations were found between SUA and LVM (*r* = 0.144, *p* = 0.155) and LVMI (*r* = 0.112, *p* = 0.269; Fig. 2) in men. In females, SUA was significantly associated with hs-CRP (*r* = 0.264, *p* = 0.038), LA diameter (*r* = 0.277, *p* = 0.029), LVM (*r* = 0.330, *p* = 0.009), and LVMI (*r* = 0.372, *p* = 0.003; Fig. 2). In addition, there were no significant correlations between SUA levels and age, blood pressures, eGFR, TC, fasting blood glucose (FBG), glycated hemoglobin (HbA1c), NT-proBNP, LVOT gradient (at rest or after provocation), MWT, or LVEF in each gender group.

**Table 3:** Correlations between serum uric acid and clinical parameters by gender

	Male (*n* = 99)		Female (*n* = 62)	
Variable	*r*	*p* value	*r*	*p* value
Age (years)	−0.03	0.768	0.095	0.463
Body mass index (kg/m^2^)	0.309	0.002	0.064	0.619
Duration of obstructive HC (months)	0.129	0.203	0.122	0.347
Systolic blood pressure (mmHg)	−0.112	0.268	0.05	0.702
Diastolic blood pressure (mmHg)	−0.053	0.601	0.013	0.922
Heart rate (beats/min)	0.015	0.881	0.124	0.338
Serum creatinine (μmol/L)	0.136	0.178	0.132	0.307
eGFR (mL/min/1.73 m^2^)	−0.065	0.524	−0.1	0.440
Fasting blood glucose (mmol/L)	0.113	0.264	0.004	0.976
Total cholesterol (mmol/L)	0.16	0.113	0.092	0.476
HDL cholesterol (mmol/L)	−0.259	0.010	0.012	0.928
LDL cholesterol (mmol/L)	0.140	0.168	0.171	0.183
Triglycerides (mmol/L)	0.343	0.001	0.129	0.319
hs-CRP (mg/L)	0.198	0.049	0.264	0.038
NT-proBNP (pmol/L)	0.049	0.633	0.162	0.208
HbA1c (%)	−0.001	0.988	0.11	0.397
LVOTG at rest (mmHg)	−0.149	0.141	−0.095	0.462
LVOTG after provocation (mmHg)^a^	0.036	0.841	−0.126	0.643
Left atrium diameter (mm)	0.247	0.014	0.277	0.029
LV end-diastolic diameter (mm)	−0.072	0.480	−0.07	0.591
Maximum wall thickness (mm)	−0.032	0.752	0.075	0.563
LV ejection fraction (%)	0.060	0.558	0.106	0.413
LV mass (g)	0.144	0.155	0.330	0.009
LV end-diastolic volume index (mL/m^2^)	−0.069	0.499	0.013	0.923
LV end-systolic volume index (mL/m^2^)	−0.097	0.341	−0.072	0.577
Stroke volume index (mL/m^2^)	−0.033	0.748	0.072	0.580
Cardiac index (L/min/m^2^)	−0.081	0.426	0.047	0.720
LV mass index (g/m^2^)	0.112	0.269	0.372	0.003

Abbreviations as in Table 1

^a^LVOTG was provoked in 50 of the 161 study patients

SUA concentrations were similar between smokers and non-smokers in both men and women (data not shown). Additionally, SUA levels did not differ between patients with and without hypertension, diabetes mellitus, or dyslipidemia, in either male or female subgroups. Likewise, the use of loop or thiazide diuretics, as well as angiotensin-converting enzyme inhibitors or angiotensin receptor blockers, did not affect SUA concentrations in both genders. In females, there were no significant differences in SUA levels between patients with menopause (*n* = 31) and those without (*n* = 31; 312.9 ± 66.9 vs. 305.1 ± 72.6 μmol/L, *p* = 0.665).

Multiple linear regression analysis was performed to determine whether the correlations between SUA and LVMI observed in females on univariate analysis were still significant after controlling for potential confounding factors affecting LVMI. In females, SUA was independently associated with LVMI (*β* = 0.375, *p* = 0.002), after adjustment for age, menopause, BMI, hypertension, diabetes mellitus, dyslipidemia, smoking, eGFR, usage of ACEI/ARB, usage of diuretics, hs-CRP, duration of obstructive HCM, and resting LVOT gradient (Table 4). In addition, resting LVOT gradient was also independently associated with LVMI in women (*β* = 0.320, *p* = 0.007). However, on multiple linear regression analysis including the same covariables (except menopause) as in females, no variables were entered into the equation for males. Moreover, replacing hypertension with systolic and diastolic blood pressures, diabetes mellitus with FBG or HbA1c, dyslipidemia with TC, TG, LDL-C, and HDL-C, and eGFR with serum creatinine in those models did not materially alter the independent associations between SUA and LVMI in women (*β* = 0.345, *p* = 0.003). Similarly, not any covariates were independently related to LVMI in men.

**Table 4:** Multiple linear regression analysis for variables associated with left ventricular mass index in women

Variable	Standardized coefficients (*β*)	*p* value
Uric acid	0.375	0.002
LVOTG at rest	0.320	0.007

Multiple *R* = 0.469, *R* ^*2*^ = 0.220, abbreviations as in Table 1

## Discussion

A large body of evidence suggests that SUA levels are significantly related to LVH and LVMI in different study populations, including patients with essential hypertension, CKD and renal transplant, and the general population. Iwashima et al. demonstrated that SUA was independently associated with LVMI in 619 hypertensive patients [21]. Moreover, they also showed that hyperuricemia combined with LVH was an independent and powerful predictor for cardiovascular disease, including myocardial infarction, angina pectoris, congestive heart failure, cerebral infarction, and transient cerebral ischemia. In a total of 540 patients with CKD, SUA was positively correlated with LVMI [23], which was further validated in female CKD patients of another study [22]. After adjustment for potential confounding factors, a significant and independent relationship between SUA and LVMI was observed in renal transplant recipients [24]. In a general population of 3305 males, the prevalence of LVH diagnosed by electrocardiography was independently associated with SUA concentration [14]. Recently, Zhu et al. reported that MWT increased significantly with ascending tertiles of SUA in patients with HCM [30]. During a mean follow-up of 5 years in that study, elevated uric acid levels independently predicted adverse outcomes of HCM. However, the association between SUA and LVMI remains unclear in patients with HCM (including obstructive HCM). In the current investigation, SUA was positively correlated with LVMI, but not with MWT, in women with obstructive HCM on univariate analysis. After adjusting for possible confounding factors which might affect LVMI, SUA was still independently associated with LVMI in those female patients. Considering that LVMI assessed by CMR was a sensitive predictor for HCM-related death [37], the significant relationship between SUA and LVMI found in this study may, at least partially, account for the independent prognostic values of SUA for adverse outcomes in patients with HCM [30].

The precise mechanisms underlying the relationship between SUA and LVMI found in this study are still undetermined. Several possible pathophysiological mechanisms are proposed as follows. First, it has been reported that SUA increases tumor necrosis factor-alpha, stimulates mitogen-activated protein kinases, and activates the renin-angiotensin system, all of which are known to promote cardiac hypertrophy [38–40]. Second, two previous studies have indicated that hyperuricemia may lead to cardiac hypertrophy by increasing arterial stiffness, which reflects the severity of LV afterload [15, 19]. Third, SUA is the final product of purine metabolism and catalyzed by xanthine oxidase (XO). SUA levels may reflect the degree of XO activity and resultant oxidative stress, which plays an important role in the development of LVH [41]. Finally, as a common inheritable heart disease, whether the association between SUA and LVMI observed in our obstructive HCM patients is dependent on cardiac sarcomere gene mutations or not remains undefined.

Previous investigations examining the sex-specific relationship between SUA and LVH yielded conflicting results. In a cohort of essential hypertension, SUA was independently associated with LVMI in both male and female patients [21]. At least three prior studies that performed sex-specific analyses demonstrated a positive association of SUA with LVMI and prevalence of LVH in female hypertensive and CKD patients, but not in their male counterparts [17, 20, 22]. In contrast, some other reports suggested that the independent association between SUA and LVH was only present in males of a general population and men with hypertension [14, 16, 18]. To date, this is the first study to explore the sex differences in the relationship between SUA and cardiac hypertrophy among obstructive HCM patients. As expected, SUA levels and LVMI were significantly higher in men than in women. LVMI increased progressively from the lower to the upper tertiles of SUA and was positively correlated with SUA concentrations only in our female obstructive HCM patients. The positive relationship between SUA and LVMI in women remained significant even after adjustment for potential confounding factors, in multivariate linear regression analysis. Although we were unable to elucidate the definite causes for this gender difference, sex hormones might play a role in this regard [16, 17, 20, 22]. It is proposed that increased renal clearance of urate related to estrogen in premenopausal women may account for the lower SUA levels observed in women than in men [20]. There was a trend toward lower SUA levels in the premenopausal women as compared with those of post-menopause in our study; however, statistical significance was not reached. In the first study to explore clinical implications of plasma UA levels on the prognosis of patients with HCM, Zhu et al. did not analyze by sex and only adjusted for sex in multivariate modeling [30]. Further studies with larger population are required to clarify the precise reasons for these gender differences regarding the relationship between SUA and LVH.

It has been proven that allopurinol, via inhibition of XO, could induce regression of LVH in humans in a wide spectrum of diseases, including CKD, ischemic heart disease, and type 2 diabetes mellitus [42–44]. The significant association between SUA levels and LVMI observed in our cohort suggests that the use of uric acid lowering drugs (e.g., allopurinol), on the basis of conventional drugs for HCM and septal reduction therapy, may theoretically result in an improvement in LV hypertrophy in patients with obstructive HCM, and subsequent amelioration of cardiovascular outcomes in those patients. Future prospective randomized placebo-controlled trials are warranted to verify that hypothesis.

Of note, contrary to findings of the aforementioned literature, several other studies have indicated a lack of association of SUA with LVH in hypertensive patients [45–47]. These discrepancies may partly be explained by methodological differences and heterogeneity of patient characteristics [45, 46]. Hence, whether the relationship between SUA and LVH in our female patients with obstructive HCM is circumstantial or causal remains to be clarified.

In accordance with a previous HCM report which found that LVMI was greater in patients with LVOT obstruction at rest [37], our multivariate analysis showed that resting LVOT gradient was independently associated with LVMI in the female subgroup. Our findings provide a further support to the hypothesis of secondary hypertrophy caused by impedance to LVOT in HCM [37].

### Study limitations

This study has several limitations. First, this was a cross-sectional and single-center study with relatively small sample size. However, by using CMR which has superior reproducibility, our study population may be large enough to verify findings in the present study. Second, we included patients who were taking loop or thiazide diuretics, which might influence SUA levels. Nevertheless, usage of these medications was associated with SUA neither in univariate nor in multivariate analysis in this study. Third, all of our subjects enrolled were from a selected population of Chinese patients with obstructive HCM, most of whom had severe dyspnea. The findings of our study cannot be extrapolated to other ethnic groups and those with non-obstructive HCM. Finally, although HCM is a common genetic cardiac disease, no genetic testing was performed in the current study. Future studies with genotyping in this population would clarify the potential effects of sarcomere mutations on the relationship between SUA and LVMI.

## Conclusions

SUA levels are significantly and independently associated with LVMI in female patients with obstructive HCM, but not in males. These findings imply that usage of urate-lowering drugs (e.g., allopurinol) to regress LVM may be a new therapeutic target in women with obstructive HCM and could provide novel opportunities to change the natural history of obstructive HCM, especially in women. Further studies are needed to confirm that hypothesis.
